# Comparative Study of the Properties of Plasticized Polylactic Acid with Maleinized Hemp Seed Oil and a Novel Maleinized Brazil Nut Seed Oil

**DOI:** 10.3390/polym13142376

**Published:** 2021-07-20

**Authors:** Aina Perez-Nakai, Alejandro Lerma-Canto, Ivan Domingez-Candela, Daniel Garcia-Garcia, Jose Miguel Ferri, Vicent Fombuena

**Affiliations:** 1 Technological Institute of Materials (ITM), Universitat Politècnica de València (UPV), Plaza Ferrándiz y Carbonell 1, 03801 Alcoy, Spain; aipena@epsa.upv.es (A.P.-N.); allercan@epsa.upv.es (A.L.-C.); dagarga4@epsa.upv.es (D.G.-G.); joferaz@upvnet.upv.es (J.M.F.); 2 Instituto de Seguridad Industrial, Radiofísica y Medioambiental (ISIRYM), Universitat Politècnica de València (UPV), Plaza Ferrándiz y Carbonell s/n, 03801 Alcoy, Spain; ivdocan@doctor.upv.es

**Keywords:** maleinized hemp seed oil, maleinized Brazil nut seed oil, bio-plasticizers, polylactic acid

## Abstract

In this study, for the first time, Brazil nut seed oil was chemically modified with maleic anhydride to obtain maleinized Brazil nut seed oil (MBNO). The same process was developed to obtain maleinized hemp seed oil (MHO). The use of MBNO and MHO was studied as bio-based plasticizers by incorporating them with different contents ranging from 0 to 10 phr in a polylactic acid (PLA) matrix. By means of mechanical, thermal and thermomechanical characterization techniques, the properties of the different formulations were studied to evaluate the plasticizing effect of the MBNO and MHO. With the addition of both plasticizers, a significant increase in ductile properties was observed, reaching an increase in elongation at break of 643% with 7.5 phr MBNO and 771% with 10 phr MHO compared to neat PLA. In addition, it has been observed that the mechanical resistant properties do not decrease, since the oils enhance the crystallization of PLA by increasing the free volume between its chains and counteracting the effect. Finally, a disintegration test was carried out under thermophilic conditions at 58 °C for 27 days, demonstrating that the incorporation of MHO and MBNO does not significantly affect the biodegradability of neat PLA.

## 1. Introduction

Since the end of the 20th century, environmental concerns and the development of sustainable strategies have been on the rise [1]. This concern carries over to the present day, where plastic represents a large part of human life, with up to 368 million tonnes of plastic produced globally in 2019 [2]. The problem arises mainly because of the negative impact on the ecosystems generated by the amount of plastic waste, since only 173 million tonnes are collected for recycling or landfill [3], but also by the exploitation of non-renewable natural resources, such as petroleum. The problem is magnified in the packaging industry, as it is the largest consumer of plastics, accounting for 39.6% of total plastic consumption in Europe [2]. In view of this situation, research is mainly focused on obtaining polymeric materials with formulations based on renewable resources or even with the property of being biodegradable. A wide variety of biopolymers or bio-based materials have now been obtained that contribute to this sustainable development.

Bio-based polymers are polymers that are made from biological substances, i.e., non-fossil materials [4]. These materials may or may not be biodegradable depending on their ability to be broken down by microorganisms [4]. Currently, some of the most promising biopolymers are extracted from biomass (starch, cellulose, protein, chitin, etc.), such as thermoplastic starches (TPSs), given the abundance of polysaccharides in the biomass. Another promising alternative are also those obtained by microbial production, such as polyhydroxyalkanoates (PHAs) and polyhydroxybutyrate (PHB) or polylactic acid (PLA) [5,6,7]. 

In 2020, bioplastics accounted for only 1.2 million tonnes, less than 1% of all plastics produced [3]. However, the market for these polymers has been growing for years and the trend is expected to increase, with production estimated to increase by 11.28% from 2019 to the end of 2025 [8]. Specifically, PLA is one of the biopolymers with the greatest potential for industrial use as a substitute for petroleum-based polymers [9,10]. This is due to its great potential due to the combination of its mechanical properties, easy processability and its price compared to other biopolymers [11,12]. PLA has a suitable thermal stability and resistance to being industrially processed by injection molding, welding, thermoforming or extrusion [13]. Moreover, as it is biodegradable, one of its most interesting applications is mainly in the packaging sector [14]. In terms of mechanical properties, this material is comparable to non-degradable polymers in the “commodity” range [14,15]. However, there is a major limitation of PLA, its brittleness, which is a major drawback in the packaging industry [16]. To alleviate this disadvantage, there are several proposals. One solution to improve the flexibility of this polymer is its blending with other polymers such as polyethylene glycol (PEG), thermoplastic starch (TPS) or polybutylene succinate-co-adipate (PBSA), among others. However, the lack of miscibility between the components makes it difficult to achieve this improvement in toughness [5,11,17,18]. For this reason, some authors proposed modified vegetable oils (MVOs) as compatibilizing agents or even as plasticizing additives [12,19]. The reason for this is that they are a renewable and sustainable substitute for synthetic modifiers [20,21], which are also respectful of human health due to their non-toxicity, as they do not generate the migration of substances such as Bisphenol A (BPA), as is the case with conventional epoxy resins used in consumer products [22]. 

Several techniques can be used for chemical modification, such as epoxidation [23,24], maleinization [5,10,25], acrylation or hydroxylation. The MVOs available on the market today are mainly epoxidized soybean oil (ESBO) [25] and epoxidized linseed oil (ELO) [26]. Authors such as Garcia-Garcia et al. or Chieng et al. [26,27] have reported the improvement of PLA stiffness properties with these oils. Additionally, other authors investigated other modified oils for PLA formulations such as epoxidized cottonseed oil (ECSO) [24] or epoxidized palm oil (EPO) [27]. Another commercial option is maleinized linseed oil, which has provided excellent properties to PLA, as reported Ferri et al. [28].

**Figure 1:**
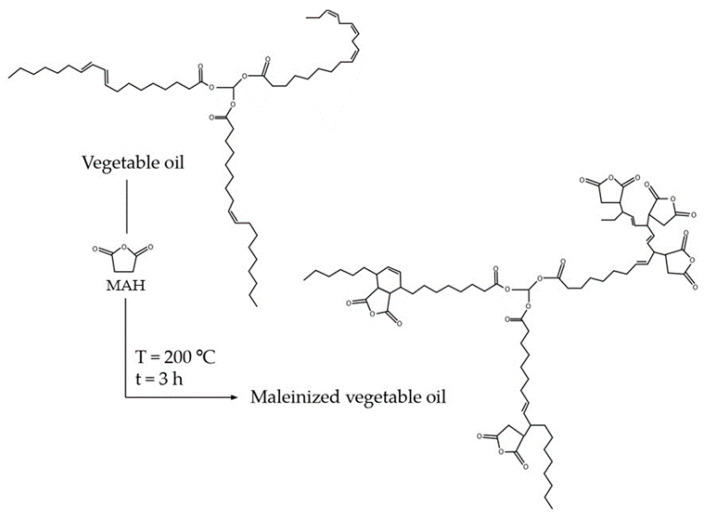
Schematic representation of the maleinization process of the triglyceride presents in vegetable oil.

In this work, the chemical modification of process of oils carried out is the maleinization. Maleinization is a chemical process, usually carried out in a single step, which consists of incorporating maleic anhydride molecules into the triglycerides that make up vegetable oils when conjugated carbon–carbon double bonds are present. For this purpose, there are several methods that can be used, such as the so-called “ene” reaction, Diels-Alder addition and free radical copolymerization, the first being the most favorable and the one used in this work [5,29]. This procedure requires a temperature of about 200 °C to result in the addition of the anhydric groups at the allylic positions of the fatty acid, as shown in Figure 1.

Both Brazil nut (*Bertholletia excelsa*) and hemp seed (*Cannabis sativa* L.) are interesting as MVOs, since both have an interesting lipid profile for functionalization. The Brazil nut is a brown fruit generally cultivated in the Amazon [30]. Its oil is an interesting object of study, since it has between 60.8% and 72.5% lipids [30,31]. Among them, it has 75.6% unsaturated fatty acids (UFA) [30], and both monounsaturated (MUFA) and polyunsaturated (PUFA), which means a significant amount of double bonds that allow its functionalization by chemical processes such as maleinization. On the other hand, hemp seed oil is also attractive for this purpose, as it is high in linoleic acid (55.3%) and linolenic acid (20.3%) [32]. Both allow their oil to be extracted by cold pressing with a good yield and also have good oxidative stability [31,33]. Thereupon, this work evaluates the potential of MBNO and MHO as a bio-based plasticizer to improve the ductile properties of PLA related to its brittleness and the comparison of these with commercial maleinized linseed oil (MLO).

## 2. Materials and Methods

### 2.1. Materials

The Brazil nuts used in this work were of Brazilian origin and were purchased from FrutoSeco (Alicante, Spain). On the other hand, the hemp seeds were obtained from a local market in Callosa de Segura (Alicante, Spain). The oil from both seeds was extracted by cold pressing with an extruder model DL-ZYJ05 purchased from Nanchang Dulong Industrial Company (Zucheng, China). The density of Brazil nut oil was 916 kg·m^−3^, its iodine value (IV) between 99.5 and 102.6 g 100 g^−1^ and its acid value (AV) 0.2 mg·KOH g^−1^. The density of hemp oil was 935 kg·m^−3^, its IV is 163.8 g·100g^−1^ and its AV is 8 mg·KOH g^−1^. For both cases, density, IV and AV values were obtained according to ISO 1675, ISO 3961 and ISO 660, respectively. For the maleinization of both oils, maleic anhydride supplied by Sigma Aldrich (Madrid, Spain), with a purity of 98% was used. The PLA used for mixing with the oils was a commercial-grade Ingeo Biopolymer 2003D from NatureWorks LLC (Minnetonka, MN, USA) in pellet form. 

### 2.2. Maleinization of Brazil Nut (Bertholletia excelsa) and Hemp (Cannabis sativa *L.*) Oils

The same method was used for the maleinization of Brazil nut and hemp seed oil. For this purpose, a round flask with three necks with a capacity of 500 mL was used, where a stirrer was placed in the central neck and used at a speed of 300 rpm. A digital thermometer was placed in the second neck to measure the temperature during the process, and the extraction of samples and the introduction of maleic anhydride was carried out through the third neck. The maleinization ratio used was 2.4:1, following recommendations from previous works [5]. A total of 9 g of maleic anhydride per 100 g of each virgin oil was introduced. During the maleinization process, three temperature stages were used, at 180 °C, 200 °C and 220 °C, and samples were taken every 30 min. Initially, 300 g of oil was introduced into the round flask and when the first temperature of 180 °C was reached, 1/3 of the quantity of the total amount of maleic anhydride was added. This temperature was maintained for 1 h and increased to repeat the same at 200 °C and 220 °C. Finally, the mixture was allowed to cool to room temperature. The degree of maleinization of the oil was determined according to ISO 660:2009 using the following expression:

(1) $$Acid\;Value=\frac{56.1\;\times \;c\;\times \;V}{m}$$

where *c* is the exact concentration of the KOH standard solution used (mol·L^−1^), *V* the volume of the KOH standard solution used (mL) and *m* the mass analyzed (g).

### 2.3. Manufacturing of PLA with Maleinized Vegetable Oils

Several PLA compositions were made with different phr of MBNO and MHO, which are summarized in Table 1, where the codes assigned to each of them are indicated. Prior to mixing, the PLA was dried for 24 h at 40 °C. The required amounts of PLA and oil were weighed and added, to be mixed manually. The mixtures were processed with a co-rotating twin-screw extruder (D = 30 mm; L/D = 20:1) from DUPRA (Alicante, Spain), at a rotation speed of 40 rpm with a temperature profile of 165 °C, 168.5 °C, 172.5 °C and 175 °C from the feed zone to die, respectively. The compositions obtained were cooled to room temperature, pelletized and dried at 40 °C for 24 h. Finally, each composition was shaped by injected molding from Mateu and Solé (Barcelona, Spain) at temperature profiles of 170 °C, 180 °C, 190 °C and 200 °C from the feed section to injection nozzle. 

### 2.4. Mechanical Characterization of PLA Formulations Plasticized with MBNO and MHO

Several standardized tensile, flexural, impact and hardness tests were carried out to determine the mechanical properties of the different PLA compositions manufactured with the different types and proportions of MVOs. The tensile and flexural tests were performed according to ISO 527 and ISO 178, respectively, with an Ibertest ELIB 30 universal testing machine from SAE Ibertest (Madrid, Spain). For the tensile test, a crosshead speed of 10 mm·min^−1^ was used with a 5 kN load cell. In the case of flexural test, the deformation rates were studied at 5 mm·min^−1^. Both tests were performed on at least 5 different samples of each material and the average values of the elongation at break (%), tensile and flexural strength, and Young’s modulus and flexural modulus were calculated. To determine the impact resistance by energy absorption, a 6 J Charpy pendulum from Metrotec S.A. (San Sebastian, Spain) was used. According to ISO 179, rectangular samples of 80 × 10 × 4 mm^3^ without notches were employed. Finally, a Shore D hardness tester model 673-D from J. Bot S.A. (Barcelona, Spain) was used to determine the hardness according to ISO 868. A minimum of 5 samples were used and the results shown are the obtained average. 

### 2.5. Thermal Analysis of PLA Formulations Plasticized with MBNO and MHO

The thermal properties of PLA and plasticized PLA compositions were determined by differential scanning calorimetry (DSC) and thermogravimetric analysis (TGA). The glass transition temperature (T_g_), cold crystallization temperature (T_cc_) and melting temperature (T_m_) were obtained by DSC with a Mettler Toledo DSC 821 (Schwerzenbach, Switzerland). The test conditions were under nitrogen atmosphere (flow rate 66 mL·min^−1^) with a thermal program to remove thermal history consisting of a first heating from 30 °C to 200 °C at 10 °C·min^−1^, followed by a cooling to 30 °C at 2 °C·min^−1^ and a last heating to 350 °C at 2 °C·min^−1^. Thermal transitions were determined from the second heating. The percentage crystallinity of each material was calculated using the following equation:

(2) $$X_{c\;}\left(\%\right)=\frac{\Delta H_{m}-\Delta H_{c}}{w\Delta H_{m}^{o}}\times 100$$

where ∆*H_m_* is the enthalpy of fusion, ∆*H_c_* the cold crystallization enthalpy, *w* the mass fraction of the material and $\Delta H_{m}^{o}$ the enthalpy of fusion for a theoretical pure crystalline PLA structure, which was assumed to be 93 J g^−1^ [34].

TGA tests were carried out to determine the initial degradation temperature (T_0_) and the maximum degradation temperature (T_max_). The equipment used was a TGA/SDTA 851 from Mettler Toledo (Schwerzenbach, Switzerland) and the samples (7–10 mg) were tested under nitrogen atmosphere (flow rate 66 mL·min^−1^) and heating from 30 °C to 700 °C at 20 °C·min^−1^. T_0_ was determined at 5% mass loss, while T_max_ was calculated from the first derivate of the TGA curves (DTG).

### 2.6. Thermomechanical Characterization of PLA Formulations Plasticized with MBNO and MHO

To analyze the changes in storage modulus (G’) and damping factor, a dynamic thermo-mechanical analysis (DMTA) was performed with an oscillatory rheometer AR G2 from TA Instruments (New Castle, DE, EEUU), equipped with a clamp attachment for solid samples. The 40 × 10 × 4 mm^3^ rectangular samples were tested in torsion mode and subjected to a thermal program from 30 °C to 130 °C with a heating rate of 2 °C·min^−1^. The frequency was set at 1 Hz and the maximum deformation (*γ*) was 0.1%.

### 2.7. Degree of Disintegration under Composting Conditions of PLA Formulations Plasticized with MBNO and MHO

The degree of disintegration of each compound was carried out according to the recommendations of the ISO 20200 standard. For this purpose, 7 different square samples for each compound of dimensions 25 × 25 × 1 mm^3^ were buried in controlled soil. Previously, samples were dried at 40 °C for 24 h. To evaluate the disintegration process, samples were unburied on days 3, 7, 14, 17, 21, 24 and 27, washed with distilled water, dried at 45 °C for 24 h and weighed in an analytical balance. The degree of disintegration was calculated with the following equation given in ISO 20200:

(3) $$D=\frac{m_{i}-m_{r}}{m_{i}}\times 100$$

where *m_i_* is the initial mass of the sample before the test and *m_r_* is the weight of the sample extracted from compost soil on different days after drying. Photographs were also taken of each sample recovered on the days indicated to also qualitatively assess the disintegration process.

## 3. Results

### 3.1. Synthesis of Maleinized Hemp Seed Oil and Brazil Nut Oil

**Figure 2:**
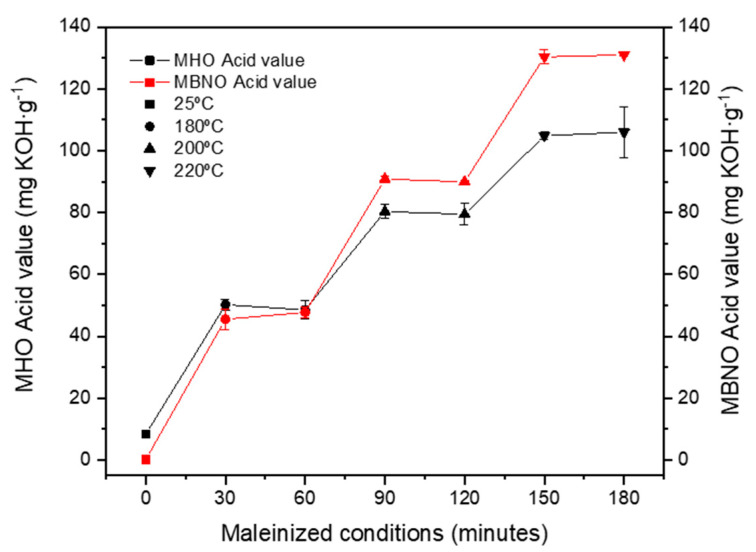
Effect of temperature and time on the efficiency of the maleinization of HO and BNO with maleic anhydride.

The evolution of the AV values along the three temperature stages of 180 °C, 200 °C and 220 °C used throughout the maleinization process of BNO and HO has been plotted in Figure 2. Initially, the AV of BNO is 0.20 mg KOH g^−1^ and 8 mg KOH g^−1^ for the HO. In both cases, it can be observed how these values increase significantly and analogously after the first hour at 180 °C to approximately 47 mg KOH g^−1^ and 50 mg KOH g^−1^ for the BNO and HO, respectively, indicating that maleinization is occurring. After two hours, at the second temperature of 200 °C, another significant increment can be observed up to values close to 90 mg KOH g^−1^ in the case of BNO and up to 80 mg KOH g^−1^ in the case of HO. Finally, at 220 °C, after three hours, there is a drastic increase in the AV up to 105 mg KOH g^−1^ in HO and even up to 130 mg KOH g^−1^ in BNO. In the two oils, the AV follows a very similar trend in which a clear increase is observed as the temperature increases. This is due to the fact that at temperatures close to 200 °C, the “ene” reaction is favored, in which maleic anhydride can easily bind to an allylic position of the unsaturated fatty acid [35]. As the temperature of 220 °C is maintained over time, the acidity index tends to stabilize, in the case of HO at 105 mg KOH g^−1^ and at 130 mg KOH g^−1^ for BNO. Both values are in complete agreement with the AV of commercial MLO reported by Quiles-Carrillo et al. [36], which indicates that it ranges between 105 and 130 mg KOH g^−1^, suggesting that both the MBNO and MHO developed have similar characteristics to one of the few maleinized oils available on the market today.

**Figure 3:**
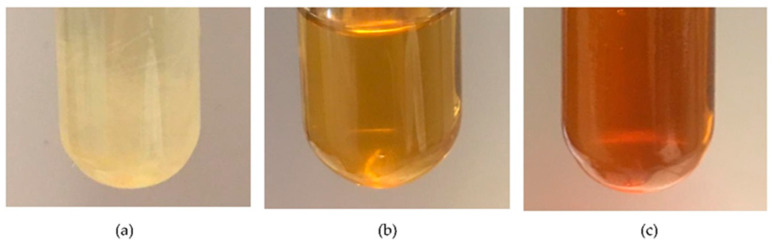
Influence of the reaction temperature and time on the color during maleinization of BNO, (**a**) 180 °C–0 min, (**b**) 200 °C–120 min and (**c**) 220 °C–180 min.

Another consequence observed as a result of the maleinization process is an important change in the color of the oils. Figure 3 shows a progressive color change of BNO, in which, at 180 °C, the color is light yellow, similar to the color of the original oil, and changes to a reddish color as maleinization occurs. The same occurs with HO. Ernzen et al. [37] reported a similar change during the maleinization process of soybean oil, in which the oil changed from a yellow to an orange–reddish color.

### 3.2. Effect of MBNO and MHO on Mechanical Properties of Plasticized PLA Formulations 

**Figure 4:**
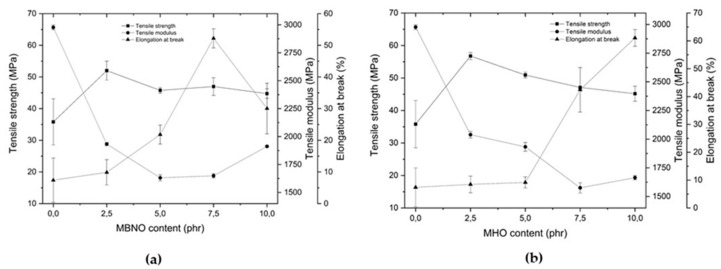
Plot of evolution of tensile properties of PLA formulations plasticized with various contents of MBNO (**a**) and MHO (**b**).

**Figure 5:**
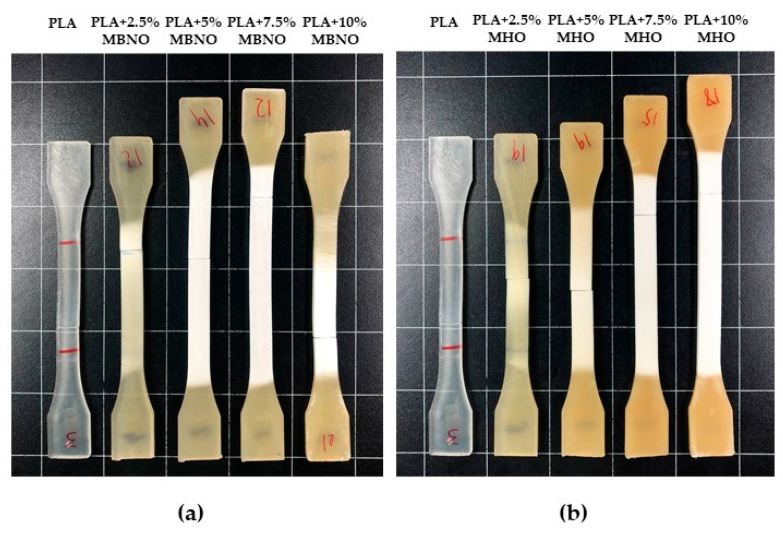
Plot of evolution of tensile properties of PLA formulations plasticized with various contents of MBNO (**a**) and MHO (**b**).

The results obtained from the mechanical properties of the different PLA formulations with both MBNO and MHO show a significant reduction in PLA stiffness, indicating that both oils are effective as renewable PLA plasticizers. Figure 4 shows the tensile mechanical properties of PLA with MBNO and MHO. Unplasticized PLA has a Young’s modulus close to 3000 MPa (2977 ± 21 MPa) and a tensile strength of 35.8 ± 7.3 MPa, relatively high values in the thermoplastic commodity range. However, its toughness is low because it shows an elongation at break of 7.4 ± 7%. By incorporating MBNO into the PLA matrix, an increase in elongation is observed from 2.5 phr, reaching the maximum value of 52% ± 3.0% at 7.5 phr, which represents an increase of 643% with respect to neat PLA. From this concentration of MBNO, a decrease in elongation occurs, probably due to plasticizer saturation, a phenomenon that some authors call an anti-plasticization effect [38]. On the other hand, the effect of MHO on the elongation at break is observed from 7.5 phr MHO where it increases drastically up to 42% ± 6% and even increases up to 61% ± 3.0% with the addition of 10 phr MHO, i.e., 771% more than neat PLA. These results obtained are close to the results reported by Ferri et al. [28], in which an elongation of 78.4% was obtained by incorporating commercial MLO into PLA. In Figure 5, it is possible to observe the changes in the appearance of the different tensile specimens after the test. Figure 5a shows specimens made with different MBNO contents. It can be seen how from 7.5 phr of MBNO content, the specimen has a lower final elongation. Figure 5b shows the specimens with different MHO contents after the tensile test. The main difference with respect to MBNO is observed in the PLA + 10% MHO sample, whose elongation is higher than that obtained with 7.5 phr MHO. This increase in elongation at break is due to the enhancement of molecular mobility, which is explained by several plasticization theories. The first of these, the lubricity theory, holds that the plasticizer functions as a molecular lubricant of the polymer. On the other hand, gel theory, applied to amorphous thermoplastics such as the grade of PLA used in this work, suggests that the plasticizer molecules are placed between the polymer chains and weaken the interactions between them. Finally, the free volume theory argues that the plasticizer increases the free volume and thus decreases the interactions between the polymer chains [39].

In addition, with the incorporation of both oils, an increase in tensile strength is observed with 2.5 phr, with respect to neat PLA, and then a decreasing trend as the percentage of oil incorporation increases. Several authors report a similar effect, but with lower strength values than PLA as an oil content increases. Garcia-Garcia et al. [40] reported a decrease in strength from 46.5 MPa to 42.2 MPa with the addition of 10% epoxidized karanja oil—EKO. In the case of MBNO and MHO, by incorporating between 2.5 and 10 phr, higher strengths were obtained than those of neat PLA, counterbalanced, in turn, by an improvement in its elongation. This phenomenon can be explained by the fact that MHO and MBNO, by improving the mobility between PLA chains, also facilitate crystallization [41], which at the same time generates an increase in its stiffness. Finally, Young’s modulus follows a decreasing trend in both cases, obtaining the lowest value with the incorporation of 5 phr MBNO of up to 45.2% and with 7.5 phr MHO of up to 47%. From these contents in phr, the modulus tends to increase as the elongation tends to decrease. This decreasing evolution is in line with the results reported by authors such as Carbonell-Verdu et al. [24], who with the incorporation of ECSO to PLA obtained a reduction in the tensile modulus of 47.5% with respect to neat PLA. The high decrease in tensile modulus obtained with MBNO and MHO is related to the decrease in molecular interactions that facilitate movement.

**Figure 6:**
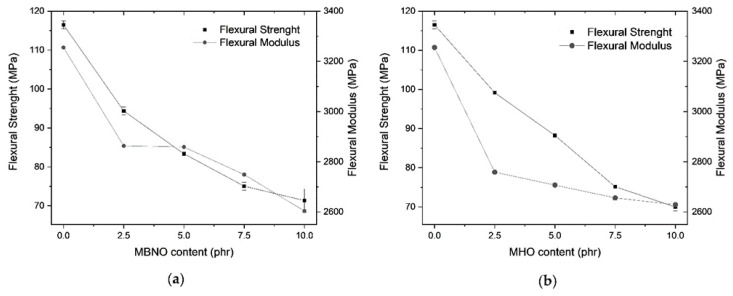
Plot of evolution of flexural properties of PLA formulations plasticized with various contents of MBNO (**a**) and MHO (**b**).

In parallel, Figure 6 shows a decrease in strength and flexural modulus as MBNO and MHO content increases. In the case of MBNO, a maximum reduction in flexural strength of 37.7% and flexural modulus of 20% is obtained with 10 phr and 39.6% and 18.4%, respectively, with 10 phr MHO content. However, as with the tensile properties, it is at 5 phr MBNO and 7.5 phr MHO that the decrease in strength and modulus tends to stabilize, suggesting plasticizer saturation. Ferri et al. [28] reported a decrease in the flexural strength of PLA with commercial MLO of up to 24%, and also a saturation around 15 phr MLO. The antiplasticizing effect of polymers depends, above all, on the molecular weight and the concentration of the diluent, so that in each formulation it is produced with a specific percentage of plasticizer [42]. 

**Figure 7:**
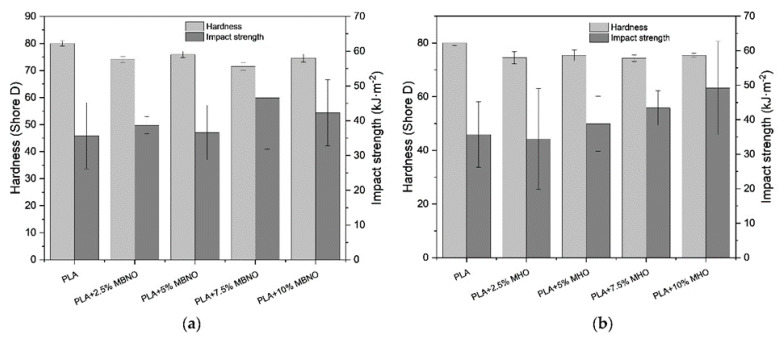
Graphic representation of impact strength and hardness (Shore D) of PLA formulations plasticized with various contents of MBNO (**a**) and MHO (**b**).

On the other hand, Figure 7 summarizes the energy absorbed by Charpy’s impact test and the Shore D hardness of neat PLA and formulations. The energy absorbed by Charpy’s impact test is closely related to the toughness of the material; therefore, it is also representative for evaluating the effectiveness of MBNO and MHO as a plasticizer. Due to its brittleness, neat PLA has a relatively low absorbed energy (around 35.5 kJ·m^−2^). By adding plasticizers, an increase in absorbed energy is observed, in the case of MBNO up to 20% higher and in the case of MHO up to 46% higher. The energy absorbed by a material depends both on the deformation capacity linked to the ductility properties and on the breaking strength, which is related to the mechanical properties [28]; therefore, the results are in full agreement with the obtained values of elongation, modulus and breaking strength. As for hardness, the value decreases progressively as more plasticizer is incorporated, although it is not as strongly visualized as with other properties. With MBNO the Shore D hardness decreased from 80 to 71.7 at 7.5 phr MBNO, and with 10 phr MHO to 72.9. Other studies reported a similar decrease from 75.6 to 59.6 with the addition of 22.5% commercial ELO to PLA/hazelnut shell flour (HSF) blends [43].

### 3.3. Effect of MBNO and MHO on Thermal Properties of Plasticized PLA Formulations

**Figure 8:**
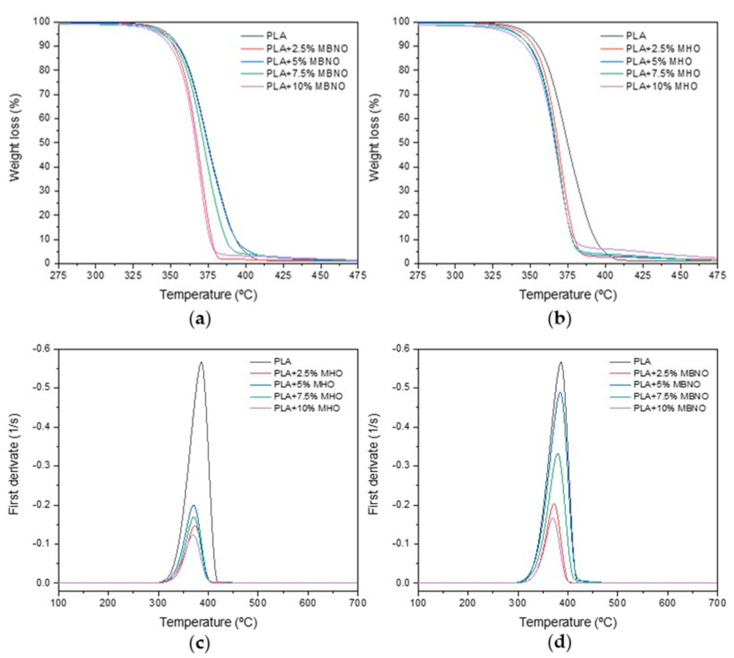
TGA (**a**,**b**) and DTGA (**c**,**d**) of unplasticized PLA and PLA plasticized with different content of MBNO and MHO.

Thermal stability of PLA and plasticized formulations with different content of MBNO and MHO was performed by thermogravimetry analysis (TGA). Figure 8 show the weight loss versus temperature curves for each sample (TG) and their corresponding first derivative curves (DGT). Table 2 shows some characteristic thermal parameters of the thermograms, such as the degradation onset temperature (T_5%_), which indicates the temperature at which a 5% weight loss occurs, and the maximum degradation temperature (T_max_), which corresponds to the peak of the first derivative curve. Neat PLA possesses good thermal stability with a T_5%_ of 347.9 °C and a T_max_ of 386 °C. As can be seen, adding MBNO or MHO to PLA causes a slight decrease in T_5%_ as they are incorporated in higher quantities. In this case, the addition of 10 phr MNBO results in a T_5%_ reduction of 4.5 °C, while with 10 phr MHO, the reduction is 10.1 °C. Regarding the maximum degradation temperature, it can be observed that both plasticizers lead to a decrease in temperature, obtaining the lowest value at 10 phr, with a reduction of 16.4 °C for both plasticizers. Similar behavior was observed by Garcia-Campo et al. [44] for PLA/PHB/PCL blends compatibilized with epoxidized soybean oil (ELO). The authors observed how the addition of ELO into the blend resulted in a reduction in T_5%_ by 19.1 °C and T_max_ by 20 °C.

**Figure 9:**
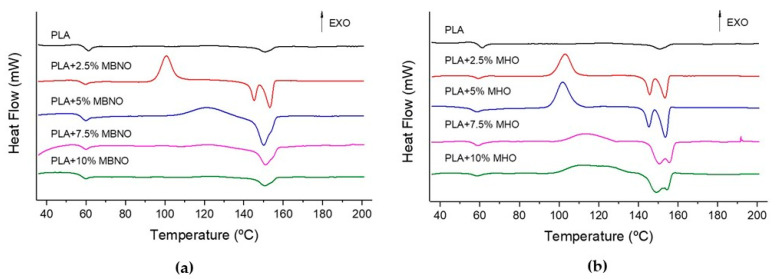
Dynamic DSC curve of PLA unplasticized and plasticized with different amounts of MBNO (**a**) and MHO content (**b**).

Table 2 shows the main thermal properties obtained by DSC. On the other hand, Figure 9 shows the plot representation of the dynamic curve of DSC obtained with the unplasticized PLA and PLA plasticized with different contents of MBNO and MHO. As can be seen, both maleinized oils have a direct effect on some thermal properties of PLA. Unplasticized PLA has a glass transition temperature (T_g_) located at 61 °C, a cold crystallization temperature (T_cc_) at 123.6 °C and a melting temperature located at 150.7 °C. Both plasticizers, MBNO and MHO, decrease the T_g_ and T_cc_ of PLA, which is indicative of an increase in the mobility of the polymer chains at lower temperatures, evidencing the plasticizing effect of both maleinized oils [45]. The same evolution was reported by Dominguez-Candela et al. [46], who employed epoxidized chia oil (ECO) in PLA. On the other hand, no significant variations were observed in T_m_, showing that after the addition of the plasticizers, the T_m_ remained at values around 150–153 °C for all the samples. As it is possible to observe in Figure 9, the addition of lower contents of maleinized oils provides two small melting temperature peaks, which are associated to the formation of two regions with different crystallinities, a result of the compatibilization process between the PLA matrix and the plasticizer. The addition of higher oil contents broadens these two peaks, producing an overlap between them. Regarding crystallinity, it is observed that PLA without plasticizing has a crystallinity of 1.7%, whereas as MBNO or MHO is added, the crystallinity increases progressively. As can be seen in Table 2, with a 10 phr MBNO, the crystallinity of PLA increases to 7.3%, while with the 10 phr MHO, it increases to 10.6%. A similar increase was reported by Carbonell-Verdu et al. [5], who observed that the addition of 10 phr commercial MLO and MCSO to PLA increased its crystallinity up to 11.6% and 19.1%, respectively. The increase in crystallinity with the addition of plasticizers, in this case MBNO and MHO, is caused by the facilitation of the laminar rearrangement of the amorphous zones of PLA, which is caused by the increased mobility of the polymer chains [47].

### 3.4. Effect of MBNO and MHO on Thermomechanical Properties of Plasticized PLA Formulations

**Figure 10:**
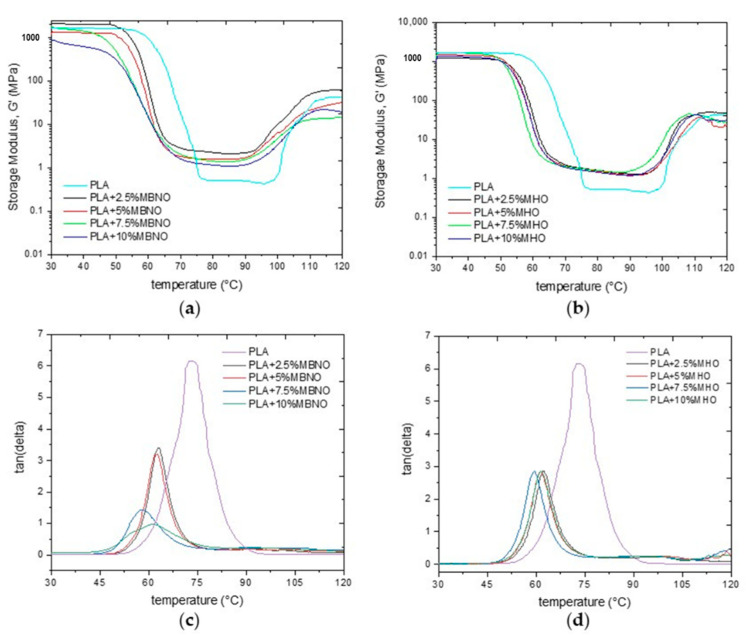
Storage modulus (**a**,**b**) and damping factor (**c**,**d**) of unplasticized PLA and PLA plasticized with different content of MBNO and MHO as a function of temperature.

The evolution of the storage modulus (G’) as a function of temperature is shown in Figure 10a,b. Neat PLA, at room temperature, has a storage modulus above 1000 MPa and this remains constant up to 60 °C. With the incorporation of MBNO and MHO in the PLA matrix, no significant changes are observed in terms of G’ at room temperature, since in all of them the values remain between 900 and 1100 MPa, although it is observed that with the incorporation of the plasticizers, the G’ tends to decrease slightly, showing its plasticizer effect. This is followed by a drop in G’ of up to three orders of magnitude, a change that is related to T_g_. In this case, a significant difference can be observed between the plasticized samples and neat PLA. As can be seen after the addition of 10 phr MBNO, the T_g_ decreases from 60 °C to 48 °C and to almost 50 °C with 7.5 phr MHO. The almost 10 °C shift in T_g_ with both plasticizers is due, as described above, to the fact that the plasticizer generates an increase in the free volume, which improves the mobility of the chain due to a lower interaction between them, being able to move with lower energy [45]. Neat PLA above 75 °C behaves like an elastomer up to 95–100 °C. Above this temperature, the energetic conditions favor and promote crystallization, improving the elastic behavior and leading to an increase in the storage modulus up to 60 MPa. Here, again, differences are observed when incorporating the plasticizers into PLA. As can be seen, this temperature decreases by around 10 °C in all the MBNO-plasticized formulations and by around 5 °C in the MHO-plasticized samples with respect to neat PLA. This decrease in temperature is due to the higher mobility of the chains, which allows their rearrangement into a packed structure with lower energy [48].

The damping factor (tan *δ*), which represents the energy lost due to viscous behavior relative to the energy stored due to elastic behavior, is shown in Figure 10c,d. The peak tan *δ*, is a way to obtain the T_g_ value of the materials and, as can be seen, while that of neat PLA is around 73 °C, in the formulations plasticized with MBNO and MHO, this temperature decreases to 58 °C and 60 °C for samples plasticized with 7.5 phr MBNO and MHO, respectively. Santos et al. [49] observed a similar decrease in T_g_ with the addition to 10 wt.% and 20 wt.% of oligoesters obtained from sunflower oil biodiesel in PLA. The authors observed that the T_g_ of PLA decreased from 62 °C to 52 °C and 44 °C, respectively.

### 3.5. Disintegration under Composting Condition of PLA Formulations

**Figure 11:**
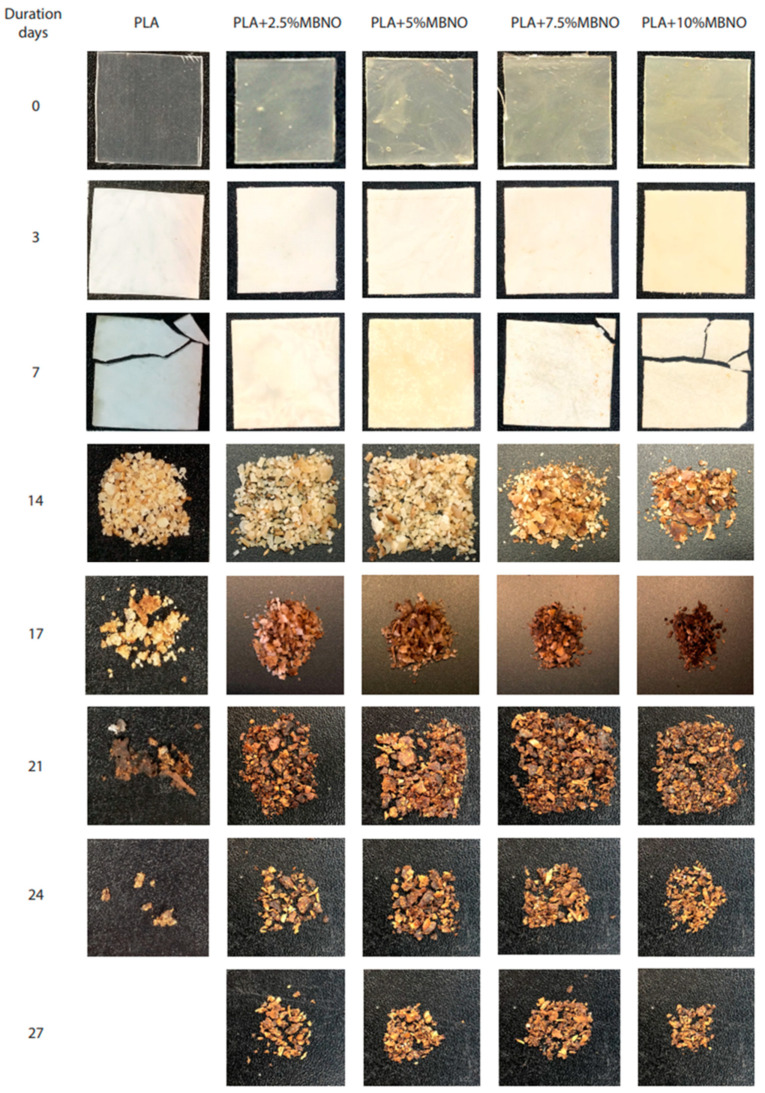
Visual appearance of disintegration under composting conditions of PLA and PLA plasticized with different contents of MBNO.

**Figure 12:**
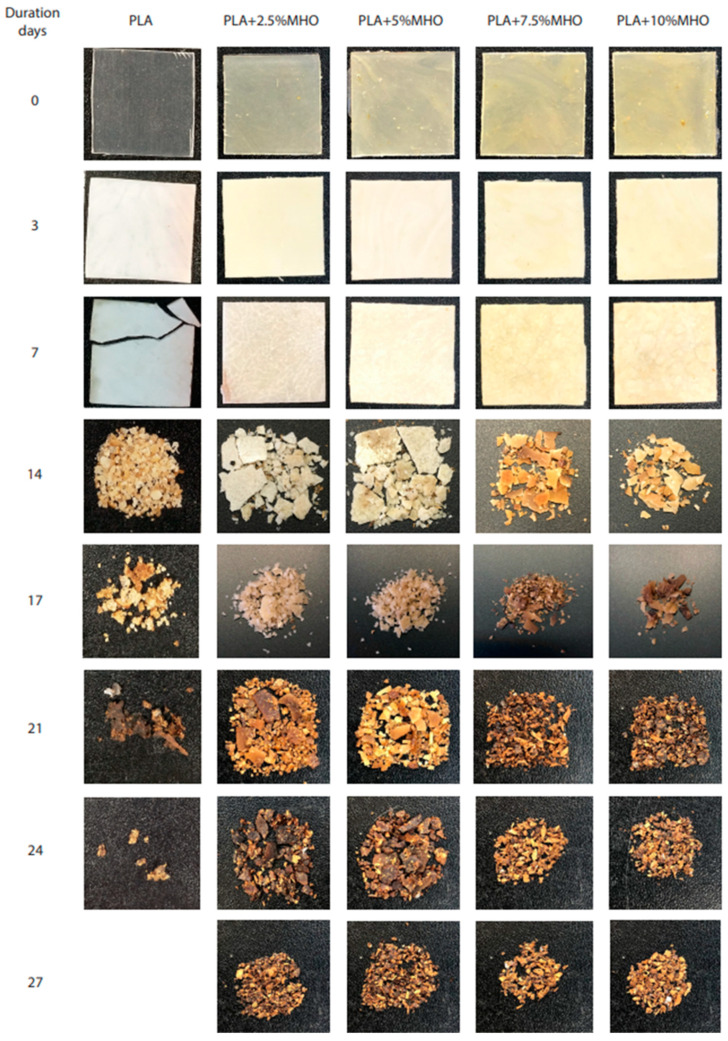
Visual appearance of disintegration under composting conditions of PLA and PLA plasticized with different contents of MHO.

**Figure 13:**
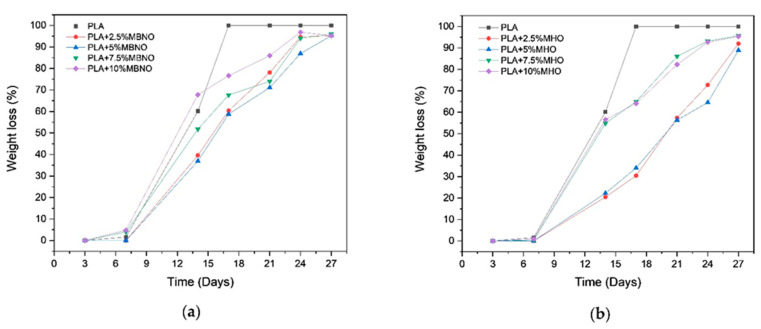
Weight loss recorded during disintegration test of PLA formulations with MBNO (**a**) and MHO (**b**).

The disintegration process of PLA formulations with MBNO and MHO in compost soil is shown visually in Figure 11 and Figure 12, respectively. Initially, the samples were translucent in all formulations; however, after 3 days of incubation, a change in visual appearance to opaque was observed. This may be due to several factors. One cause can be attributed to the 50% relative humidity test condition, since possible hydrolytic degradation due to water absorption affects the refractive index [50]. On the other hand, taking into account the fact that the test was carried out under thermophilic conditions at 58 °C, the opacity may also be due to crystallization, since this temperature is close to the T_g_ obtained in the thermal study by DSC. As can be seen in Figure 13, where the weight loss with respect to the initial mass at different periods of incubation for two PLA formulations with MBNO (a) and MHO (b) after 7 days was buried, the samples began to lose mass, which led to increased embrittlement, which is observed in the images with the appearance of cracks. However, it was not until day 14 that significant weight loss and inconsistency of the samples was observed. In the case of neat PLA, a faster degradation than in the plasticized PLA was observed, since on day 17 it had already exceeded 90% mass loss, the degree of disintegration determined by the ISO 20200 standard for considering a material to be disintegrable. In the case of plasticized PLA, in the formulations with 2.5 phr and 5 phr of both MBNO and MHO, 90% mass loss was reached at day 27, while with the 7.5 phr and 10 phr formulations, it was reached at day 24. Although the difference in the disintegration time of PLA formulations with plasticizer is small, and in all cases, the time is longer than with neat PLA, and a slight increase in the disintegration rate is observed when more plasticizer is added. This delay in disintegration with respect to neat PLA when introducing plasticizer is due to the fact that the PLA grade used in this work is very amorphous, as can be seen in the thermal analysis; when introducing plasticizer, crystallinity increases, making it difficult for microorganisms to act in the degradation, which act faster in amorphous domains [51,52]. A similar trend was reported by Balart et al. [53], who observed an increase in disintegration time upon the incorporation of ELO into the PLA matrix. Therefore, in view of the results, as a conclusion of the disintegration study, it has been demonstrated that the maleinized oils slightly retard the disintegration of PLA samples. However, PLA compounds plasticized with MBNO and MHO can be considered equally biodegradable by composting.

## 4. Conclusions

This research work has developed for the first time a maleinized Brazil nut oil (MBNO). This, as well as a maleinized hemp oil (MHO), were introduced into the PLA matrix to study and analyze their effect as bio-based plasticizers. In addition, the results obtained show MBNO and MHO provided a similar performance compared with a commercially available maleinized linseed oil (MLO), demonstrating its potential. For example, the elongation at break of neat PLA is 7.4%, which is quite low given its brittleness. With the addition of MBNO and MHO, an improvement of 643% and 771%, respectively, was achieved. On the other hand, with values higher than 7.5 phr MBNO, a decrease in the elongation at break was observed due to the anti-plasticizing effect produced by saturation in the mixture. In terms of absorbed impact energy, a 20% higher value was obtained with the addition of 7.5 phr MBNO and 46% higher with 10 phr MHO compared to unplasticized PLA. In addition, mechanical properties such as tensile strength do not decrease, as both maleinized oils provide an improvement in the mobility of PLA chains and an increase in free volume, increasing the degree of crystallinity and thus counteracting the plasticizing effect that would decrease these properties. Thermal parameters, such as T_g_, show a decreasing influence with the presence of these bio-plasticizers developed, but not drastically. Finally, the disintegration test under composting conditions showed that the addition of MBNO and MHO, although slightly delaying the process, did not lead to a loss of the biodegradability of PLA. Therefore, both MBNO and MHO are shown as potential bio-plasticizers of organic origin to increase the ductility of PLA without affecting its mechanical properties and not affecting its biodegradability, which makes them two bio-plasticizers of interest for industrial applications. 

## Figures and Tables

**Table 1:** Composition of plasticized PLA with different contents (phr) of maleinized vegetable oils and labeling of the formulations.

Reference	PLA	Maleinized Brazil Nut Oil—MBNO (phr)	Maleinized Hemp Seed Oil—MHO (phr)
PLA	100	0	0
PLA + 2.5 MBNO	100	2.5	0
PLA + 5 MBNO	100	5	0
PLA + 7.5 MBNO	100	7.5	0
PLA + 10 MBNO	100	10	0
PLA + 2.5 MHO	100	0	2.5
PLA + 5 MHO	100	0	5
PLA + 7.5 MHO	100	0	7.5
PLA + 10 MHO	100	0	10

**Table 2:** Summary of the TGA and DSC thermal parameters of PLA unplasticized and plasticized with different amounts of MBNO and MHO content.

Samples	TGA Parameters		DSC Parameters					
	T_5%_ (°C) [a]	T_max_ (°C)	T_g_ (°C)	T_cc_ (°C)	ΔH_c_ (Jg^−1^)	T_m_ (°C)	AH_m_ (Jg^−1^)	X_PLA_ (%)
PLA	347.9	386.2	61.0	123.64	1.2	150.7	3.2	1.7
PLA + 2.5%MBNO	346.6	372.1	58.0	100.8	24.9	153.3	26.8	2.1
PLA + 5%MBNO	346.5	384.6	57.9	120.8	9.2	150.3	15.0	5.8
PLA + 7.5%MBNO	344.5	379.3	58.7	123.7	3.3	151.2	10.0	6.8
PLA + 10%MBNO	343.4	369.6	57.8	123.5	2.6	151.0	3.2	7.3
PLA + 2.5%MHO	346.9	373.1	57.8	103.1	21.0	153.3	22.6	1.6
PLA + 5% MHO	342.9	370.6	56.8	101.8	20.8	153.6	28.4	7.5
PLA + 7.5% MHO	342.2	370.6	57.3	113.9	13.5	150.5	19.5	6.0
PLA + 10% MHO	337.8	369.6	56.9	109.9	9.7	149.2	20.3	10.6

[a] T_5%_, calculated at 5% mass loss.
